# Quantitative Flow Cytometric Evaluation of Oxidative Stress and Mitochondrial Impairment in RAW 264.7 Macrophages after Exposure to Pristine, Acid Functionalized, or Annealed Carbon Nanotubes

**DOI:** 10.3390/nano10020319

**Published:** 2020-02-13

**Authors:** Odile Sabido, Agathe Figarol, Jean-Philippe Klein, Valérie Bin, Valérie Forest, Jérémie Pourchez, Bice Fubini, Michèle Cottier, Maura Tomatis, Delphine Boudard

**Affiliations:** 1 Inserm U1059 SAINBIOSE, équipe DVH/PIB, Université Jean Monnet, Faculté de Médecine, F-42270 Saint-Etienne, France; 2 Université Lyon, F-42270 Saint-Etienne, France; 3 Centre Commun de Cytométrie en Flux, F-42270 Saint-Etienne, France; 4 Ecole Nationale Supérieure des Mines, SPIN, CNRS: UMR 5307, LGF, F-42023 Saint-Etienne, France; 5 Mines Saint-Etienne, Univ Lyon, Univ Jean Monnet, INSERM, U1059 Sainbiose, Centre CIS, F-42023 Saint-Etienne, France; 6 Dipartimento di Chimica and ‘G. Scansetti’ Interdepartmental Center for Studies on Asbestos and other Toxic Particulates, Università di Torino, 10125, Torino, Italy

**Keywords:** carbon nanotubes, acid functionalization, annealing treatment, oxidative stress, mitochondrial membrane potential collapse, cytotoxicity, chromatin decondensation, scavenging capacity, flow cytometry and spin trapping in-cell free system

## Abstract

Conventional nanotoxicological assays are subjected to various interferences with nanoparticles and especially carbon nanotubes. A multiparametric flow cytometry (FCM) methodology was developed here as an alternative to quantify oxidative stress, mitochondrial impairment, and later cytotoxic and genotoxic events. The experiments were conducted on RAW264.7 macrophages, exposed for 90 min or 24 h-exposure with three types of multiwalled carbon nanotubes (MWCNTs): pristine (Nanocyl™ CNT), acid functionalized (CNTf), or annealed treatment (CNTa). An original combination of reactive oxygen species (ROS) probes allowed the simultaneous quantifications of broad-spectrum ROS, superoxide anion (O_2_^•−^), and hydroxyl radical (•OH). All MWCNTs types induced a slight increase of broad ROS levels regardless of earlier antioxidant catalase activity. CNTf strongly stimulated the O_2_^•−^ production. The •OH production was downregulated for all MWCNTs due to their scavenging capacity. The latter was quantified in a cell-free system by electron paramagnetic resonance spectroscopy (EPR). Further FCM-based assessment revealed early biological damages with a mitochondrial membrane potential collapse, followed by late cytotoxicity with chromatin decondensation. The combined evaluation by FCM analysis and cell-free techniques led to a better understanding of the impacts of MWCNTs surface treatments on the oxidative stress and related biological response.

## 1. Introduction

The use of nanomaterials in many industrial fields has tremendously raised over the last two decades; thus, nanotechnologies represent a massive worldwide investment [1,2]. Carbon nanotubes present unique properties in terms of strength, electrical conductivity, or heat conduction. Therefore, they are suitable for applications in high-performance materials and recently considered for use in biomedical research [3,4,5,6,7,8]. In regard to this extensive development, the question of the risk assessment on human health is critical [9,10,11]. Carbon nanotubes are generally classified based on the number of their graphene walls. Multiwalled carbon nanotubes (MWCNTs) are thicker, usually longer, and more rigid than the single-walled (SWCNTs) or double-walled ones [12,13,14]. A number of studies have underlined that the physicochemical characteristics of MWCNTs are of paramount importance in their potential toxicity: chemical composition, shape, size, aggregation, surface charge, presence of metallic impurities and structural defects, and hydrophobicity/hydrophilicity, all seem to play a role [12,15,16,17,18,19,20,21]. In the last decade, the number of in vitro and in vivo nanotoxicological investigations have considerably increased to evaluate the potential bio-toxicity, bio-distribution, and bio-persistence of MWCNTs [11,12,15,22,23]. Despite partially conflicting results, there is growing evidence concerning the induction by MWCNTs of three main toxicological mechanisms: oxidative stress, inflammation, and cytotoxicity/genotoxicity [5,12,21,23,24,25,26]. MWCNTs seem to set off an oxidative stress associated to reactive oxygen species/reactive nitric species (ROS/RNS) production as an initial response [5,6,12,16,23,27,28]. In turn, oxidative stress triggers and maintains inflammation, and it eventually induces cytotoxic and genotoxic effects [6,12,25,28,29]. The overproduction of ROS can effectively ultimately affect biological structures and lead to cell function damages [6,16,30]. 

Metallic iron impurities resulting from MWCNTs catalysis can enhance ROS formation, especially the production of hydroxyl radical (•OH), due to the involvement of ferrous and ferric ions (Fe^2+^ and Fe^3+^) in the Fenton and Haber–Weiss cycle reactions (Figures S1 and S2) [6,16,31,32,33,34]. Hydrogen peroxide (H_2_O_2_), the other reactive of the Fenton reaction (Fe^2+^ + H_2_O_2_ → Fe^3+^ + OH^−^ + •OH), is produced by mitochondrial electron transport chains. •OH is considered as the most harmful radical species as its production can lead to mitochondrial dysfunction, and critical damages on molecular organic substrates such as proteins, lipids, and nuclear acids [6,16,30]. A review of the current literature revealed that MWCNT could induce cell death through different pathways such as apoptosis, necrosis, or autophagy mechanisms, depending on the cell model as well as MWCNTs physicochemical properties and exposure conditions [5,23,27,28,35,36,37,38,39,40]. 

MWCNTs’ physicochemical properties can be chemically modified in order to improve their biocompatibility and biodegradation, reducing their intrinsic toxicity [41,42,43,44]. In this “safer by design” approach, acid functionalization represents a major post-treatment of MWCNTs [17,20,43,45]. It purifies the MWCNTs from metallic catalytic impurities and introduces acid surface groups to decrease the MWCNTs hydrophobicity, promoting a better aqueous dispersion. However, it also generates numerous structural defects. On the other hand, annealing treatment of MWCNTs at high temperature (>1600 °C under controlled atmosphere) decreases the MWCNTs structural defects, while removing metallic impurities [43,46,47]. Nevertheless, several studies have demonstrated that these post-treatments did not necessarily reduce MWCNTs’ toxicity [20,36,37,43,45,47,48]. Then, the MWCNTs post-treatment effects on biological response are still to be fully understood, especially their impacts on oxidative stress. To our knowledge, no simultaneous comparative studies of the impact of acid functionalization and thermal annealing of MWCNTs has been published, aside from our previous work [43,47,49]. Three different MWCNTs types will be studied here: pristine MWCNTs (Nanocyl NC7000^TM^, hereafter referred simply as CNT), MWCNTs functionalized with acid groups (CNTf), and annealed MWCNTs (CNTa).

Variable and sometimes conflicting data on MWCNTs toxicity can be found in the literature due to the absence of an international standardized methodology for nanotoxicology [11,12,41,50,51]. On one hand, differences in observed toxicity may be due to the diversity of in vitro cell models, time and exposure doses, or the variability of the MWCNTs types, with sometimes incomplete physicochemical characterization to distinguish them. On the other hand, artefactual biases can skew conventional toxicological assays [12,51,52,53,54,55]. MWCNTs were indeed found to adsorb molecular reagents and culture supernatant molecules or directly interfere with optical colorimetric or fluorimetric assays, leading to misinterpretations. This has been observed for MTT ((3-(4,5dimethylthiazol-2-yl)-2,5-diphenyltetrazoliumbromide)) assay, XTT ((2,3-bis-(2-methoxy-4-nitro-5-sulfophenyl)-2H-tetrazolium-5-carboxanilide) assay, or LDH (lactate dehydrogenase) or cytokine quantifications [52,54,55,56,57,58]. Corrections of these artifacts are necessary to ensure accurate results [43,47,54,55]. Another drawback of these tests is the direct analysis of a whole cell population in their culture wells, without distinction between live and dead cells. Cellular debris can act as artefactual objects, leading to the poor performances of vital probes, and it may distort the quantitation of toxicological outcomes [59,60,61]. In this context, flow cytometry (FCM) appears as a powerful alternative methodology to perform bio-toxicological assays avoiding as much as possible any residual presence of cell debris and MWCNTs aggregates that could interfere, and at the same time discriminating cell subpopulations of interest [12,25,62,63,64,65]. We developed here a quantitative multiparametric FCM approach to investigate early alterations such as oxidative stress generation and mitochondrial potential alteration, and late events such as cytotoxicity (cell death rate) and genotoxicity (apoptotic DNA fragmentation and chromatin decondensation). Oxidative stress was extensively studied based on an original combination of three fluorescent ROS probes: DCFH-DA (2′,7′dichlorodihydrofluorescein) for the quantification of broad-spectrum ROS [28,66,67,68], HE (hydroethidine) for the detection of the superoxide anion (O_2_^•−^) [31,69,70,71], and CellRox Green for the preferential assessment of hydroxyl radical (•OH) [69,70]. A complementary analysis of anti-oxidant defense processes with superoxide dismutase (SOD) and catalase activities was conducted [6,16,30,31]. Additionally to FCM, electron paramagnetic resonance (EPR) analysis in a cell-free system was used to assess the intrinsic MWCNTs scavenging capacity: the MWCNTs potency to adsorb ROS at their surface and limit their reactivity [46,72,73].

MWCNTs can easily form aerosols, causing potential inhalation exposure [18,74,75]. Therefore, alveolar macrophages represent a preferential cell target. They are the first immune defense cells encountered by MWCNTs after inhalation, and their phagocytosis functions may trigger oxidative stress and inflammatory response [25,31,35]. RAW 264.7 macrophage cells, a referent in vitro model for nanotoxicological studies, were chosen for this study [26,31,36,38,48]. Previous work in our research group used this same cell line, which allowed to converge conclusions from several studies [43,47,49]. 

RAW 264.7 macrophages will be exposed finally to concentrations of 15 to 120 μg·mL^−1^ (within the range of commonly used MWCNTs doses [20,38,43,47,48] of pristine CNT, CNTf, and CNTa for 90 min or 24 h before complementary FCM and cell-free system analyses as previously described.

## 2. Materials and Methods

### 2.1. Cell Culture of RAW 264.7 Macrophages

RAW 264.7 murine macrophage cell line was provided by ATCC Cell Biology Collection (Promochem LGC). It derived from mice peritoneal macrophages transformed by the Albeson Murine Leukemia Virus. Cells were grown in 10% fetal calf serum Dulbecco’s Modified Eagle Medium (DMEM, Invitrogen) supplemented with 1% penicillin-streptomycin (Sigma-Aldrich) and maintained at 37 °C under a 5% carbon dioxide humidified atmosphere.

### 2.2. MWCNTs Powders Characteristics

In this study, three types of MWCNTs were used:

- CNT: pristine MWCNTs (NC7000^TM^, Nanocyl), with a diameter of 9.5 nm and lengths from 1.5 to 2 µm according to the manufacturer.
- CNTa: same MWCNTs after an annealing treatment over 2125 °C for 1 h under argon, full procedure described in Figarol et al. [47].
- CNTf: same MWCNTs after an acid functionalization by oxidation with refluxing in a solution of nitric and sulfuric acids (3:1 *v*/*v* during 6 h), as described in Figarol et al. [43].
- MWCNTs suspensions preparation and physicochemical characterizations were carried out according to a methodology detailed in our previous studies [43,47,49]. The main physicochemical features of the three MWCNTs batches (CNT, CNTf, and CNTa) are summarized in Table 1. All samples showed similar diameters and lengths. Traces of iron, a metallic catalytic impurity, were found in pristine CNT. Its level was dramatically reduced after acid functionalization or annealing treatment. Acid functionalization increased the amount of structural defects established with Raman spectroscopy (Id/Ig ratio: corresponding to the intensity of the D- to G-band ratio from the Raman spectra, respectively linked to sp^3^ and sp^2^ carbons), while annealing has the reverse effect. MWCNTs suspensions in culture medium at 37°C were stable over 48 h [43,47,49].

### 2.3. Experimental In Vitro Exposure Conditions

Three independent experiences were performed for each condition. RAW 264.7 cells were exposed to 15 or 120 µg·mL^−1^ of one type of MWCNTs in culture medium for 90 min or 24 h. A negative control (unexposed RAW 264.7 cells) was included for all experiments.

### 2.4. Flow Cytometry Toxicity Evaluation

Selection of viable cells was carried out by sequential gating using FCM analysis, according first, to Forward Scattered Light (FSC) versus Side Scattered Light (SSC) parameters, and secondly by propidium iodide (PI) staining (DNA intercalant with passive diffusion across cell membrane). This classical gating strategy is based on the optical signals (FSC/SSC) collected by the cytometer. Their respective intensity is correlated with the particulate properties whatever their nature [59]. It permits the exclusion of smaller elements such as cellular debris [60,61] and any type of aggregates [76]. In addition, by using the properties of PI as a DNA intercalant, we succeed in separating three subpopulations of interest i.e., viable cells, moribund, and dead cells. This gating strategy was illustrated in Figure S3.

#### 2.4.1. Quantitative Analysis of Radical Species 

After exposure to MWCNTS, cells were incubated with three different permeant probes, each one specific of various ROS/RNS. The well-known probe 2′,7′-Dichlorodihydrofluorescein, diacetate (DCFH-DA, Ex: 504 nm/Em: 529 nm) was used at 8 μM to quantify a broad spectrum of ROS activity (•OH, ONOO- and weaker peroxyl ROO• and H_2_O_2_ reactivity) [28,66,67,68,69]. Hydroethidine (HE, Ex: 518 nm/Em: 605 nm) preferentially reacts with the superoxide anion O_2_^•−^ [31,69,70,71], and it was used here at a final concentration of 5 μM. Finally, CellROX green (Ex: 485 nm/Em: 520 nm), a fluorogenic probe described as more specific to hydroxyl •OH radicals and exhibiting a better selectivity than CellROX deep red or CellROX orange [69,70], was used at 5 μM final concentration. 

Then, 20,000 cells were analyzed for each sample. HE exhibited a large overlay of reemission spectrum with PI. In this case, dead cells could thus be excluded only based on morphological FSC versus SSC parameters, and the quantification of radical species analyzed on the whole healthy population. Green fluorescence was displayed as univariate histogram. All results were expressed as a MFI (mean of fluorescence intensity) ratio of MWCNTs treated versus non-treated cells. All flow cytometry experiments were conducted on a FACS DiVa (BD biosciences) equipped with an argon ion and a He-Ne laser. Data were analyzed with DiVa 5.03 software.

#### 2.4.2. Cytotoxicity Evaluation: PI Permeability Versus Mitochondrial Membrane Potential (∆Ψm)

According to the introductive cytometry description, the death rate was assessed by the gradation of PI staining leading to the discrimination of the three populations of interest: viable healthy cells (PI-), intermediate moribund cells (PI+), and dead cells (PI++) compared to unexposed control cells (Figure S3). Then, a bi-parametric quantification of PI and DiOC6 (3,3′-dihexyloxacarbocyanine iodide) was realized to evaluate the mitochondrial respiration of each cell subpopulation [77]. The fluorescent probe DiOC6 accumulates in mitochondria due to the negative charge of their inner membrane when used at low concentrations of 15 nM in our experimental conditions (<100 nM) [78].

When the mitochondria respiratory chain is altered, a decrease of DiOC6 uptake is observed. This allows for the calculation of a mitochondrial membrane potential (∆Ψm). Finally, a ratio of DiOC6 MFI of MWCNTs treated cells versus untreated control was established on viable and moribund gated cells. Then, 25 µM of CCCP (carbonylcyanide-m-chlorophenyl hydrazine), a decoupling agent of the mitochondrial respiratory chain were used as positive control. A total of 20,000 cells were analyzed for each sample. 

#### 2.4.3. Genotoxicological Evaluation: DNA Strand Breaks and Chromatin Decondensation

Nuclear genotoxicity is a regular process in apoptosis. It was assessed here by the evaluation of the internucleosomal DNA (deoxyribonucleic acid) fragmentation and the chromatin condensation shape. DNA fragmentation was quantified using a TUNEL assay kit (Terminal deoxynucleotidyl transferase dUTP nick end labeling, APO-BrdU TUNEL assay kit, Molecular Probes). A fixed lymphoma cell line treated with camptothecine was used as positive control as recommended by the manufacturer. Counterstaining DNA with PI leads to simultaneous analysis of the chromatin stability. PI DNA stainability is indeed directly proportional to double-strand DNA accessibility and chromatin decondensation, which is an abnormal state of DNA conformation consecutive to genotoxic effects [59,79]. Increases in chromatin decondensation were detected by higher coefficients of variation (CV) of the G0/G1 peak in cell cycle analysis. 

### 2.5. Analysis of Superoxide Dismutase (SOD) and Catalase Anti-Oxidant Activities 

ROS are damaging when their concentrations unbalance the anti-oxidant systems. SOD and catalase are of particular importance for the regulation of respectively superoxide anion O_2_^•−^ and hydrogen peroxide H_2_O_2_ (Figure S1) [6,16,30]. SOD activity was measured on culture supernatants thanks to the Oxiselect Superoxide Dismutase Activity colorimetric Assay (Cells Biolab). In the presence of SOD, the levels of O_2_^•−^ radicals are reduced, yielding less colorimetric signal (assay reagent absorbance at 490 nm). SOD amount was quantified in regard to an established standard curve. Results were expressed in U.mL^−1^ of SOD activity.

Catalase is an antioxidant enzyme with an action rather targeted on H_2_O_2_ in order to produce water and oxygen molecules. The catalase activity assay is based on the oxidation of the non-fluorescent probe Amplex Red in the presence of H_2_O_2_ to produce resorufin, which is a fluorescent compound (Amplex Red Catalase Assay kit, Life Technologies). H_2_O_2_ levels decrease proportionally to catalase activity and lead to the production of the fluorescent resorufin (Ex: 530 nm/Em: 590 nm, read with Fluoroskan Ascent, Thermo Fisher Scientific). The catalase amount was quantified using an established standard curve and results were expressed in U.mL^−1^ of catalase activity.

### 2.6. Cell Free System

#### 2.6.1. Analysis of Bioavailable Iron Impurities Accessibility on MWCNTs Surface

Samples (0.5 mg·mL^−1^) were suspended in a solution of ferrozine (1 mM), which allows Fe^2+^ detection, or a solution of ferrozine (1 mM) containing ascorbic acid (1 mM) to detect both Fe^2+^ and Fe^3+^ (total iron), and stirred for 24 h at 37 °C. At regular time intervals (90 min, 8 h and 24 h), aliquots of the suspension were taken and centrifuged at 9000× *g* for 15 min. The amount of iron present in the supernatant was determined spectrophotometrically on a Uvikon 930 dual beam spectrophotometer (Kontron Instrument) by measuring the absorption of the iron–ferrozine complex at 562 nm. The experiments were performed in duplicate and data are presented as average values (nmol.mg^−1^) ± standard deviation (SD).

#### 2.6.2. MWCNTs Hydroxyl Radical Scavenging Activity Analysis

The analysis of MWCNTs scavenging activity was performed by generating •OH through Fenton reaction and measuring the decrease in the intensity of the EPR signal in the presence of MWCNTs. •OH radicals were generated by adding H_2_O_2_ (0.2 M) to a solution of 0.075 M of DMPO (5-5′-dimethyl-1-pirroline-Noxide) in phosphate buffer (0.125 M, pH 7.4), containing 3.2 mM FeSO_4_ and 1.25% sodium dodecyl sulfate (SDS). The amount of •OH released was monitored by measuring the intensity of the spectrum of DMPO/•OH adducts after 5, 10, 20 and 30 min of incubation by EPR spectroscopy (Miniscope 100 EPR spectrometer, Magnettech). The test was repeated in the presence of the MWCNTs samples (2 mg·mL^−1^). A nanosized amorphous silica (Aerosil 300, unable to generate radicals) was used as negative control to verify that there were no interferences. The instrument settings were as follows: microwave power 10 mW; modulation 1000 mG; scan range 120 G; center of field 3345 G. The tests were repeated at least twice with each type of MWCNTs. The •OH amount was proportional to the intensity of the EPR signal. The signals were double integrated, and the intensities were reported as average value ± standard deviation, according to previous studies [46,72,80].

### 2.7. Statistical Analysis

Statistical significance was declared when *p* < 0.05 using a Student’s test for FCM chromatin decondensation. All others FCM results were analyzed using GraphPad Prism^®^ 5 software with a two-way analysis ANOVA test with Bonferroni post hoc test, in order to perform multiple comparisons between the different MWCNTs exposure conditions and the negative control (unexposed cells). Results were expressed as the mean of three independent experiments. Data on scavenging activity (cell free system) were analyzed by a one-way Analysis of Variance (ANOVA) followed by Tukey’s post hoc test (software: SPSS 19.0 for Windows, SPSS Inc., Chicago, IL). In all cases, we have considered three levels of significance according to the *p*-value: * *p* ≤ 0.05, ** *p* ≤ 0.01, and *** *p* ≤ 0.001.

## 3. Results

### 3.1. Multi-Sided Quantification of Oxidative Stress by FCM 

We quantified first the total ROS and then the specific O_2_^•−^ and •OH productions in viable cells after PI exclusion.

**Figure 1:**
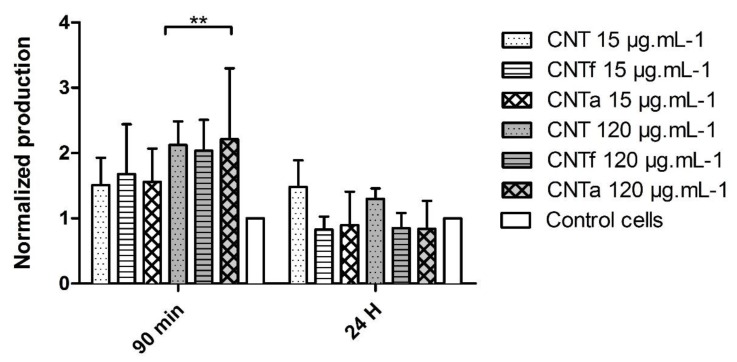
DCFH-DA (2′,7′dichlorodihydrofluorescein) cytometric quantification with reactive oxygen species (ROS) broad-spectrum production expressed as a normalized ratio of the mean of fluorescence of treated versus untreated cells. Treatments implied three categories of multiwalled carbon nanotubes (MWCNTs) (pristine or Nanocyl™ CNT (CNT), acid functionalized (CNTf), and annealed treatment (CNTa)) at 15 μg·mL^−1^ and 120 μg·mL^−1^, after 90 min and 24 h exposure (** *p* ≤ 0.01, ANOVA test, comparison to the negative control). The DCFH-DA reactivity has been verified with a positive H_2_O_2_ control (data not shown).

For the DCFH-DA quantification (method of reference, Figure 1) after 90 min, the levels of intracellular ROS in the cells exposed to a 120 µg·mL^−1^ dose increased by twice with respect to the negative control, without distinction of MWCNTs types. After long exposure (24 h), ROS level tended to lower compared to the acute exposure time (90 min) and returned to values similar to the negative control. 

**Figure 2:**
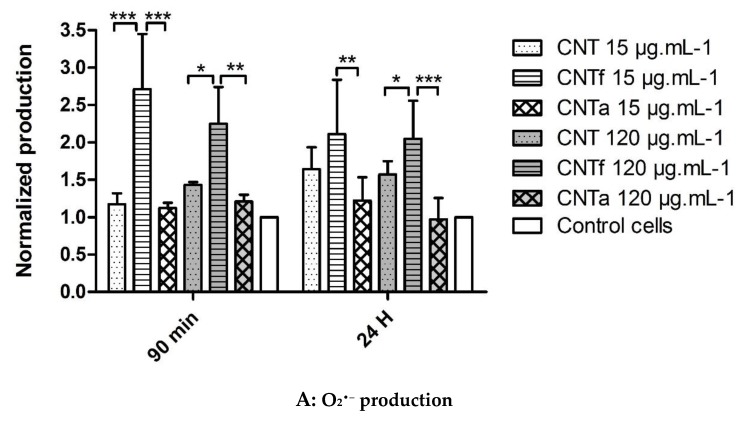

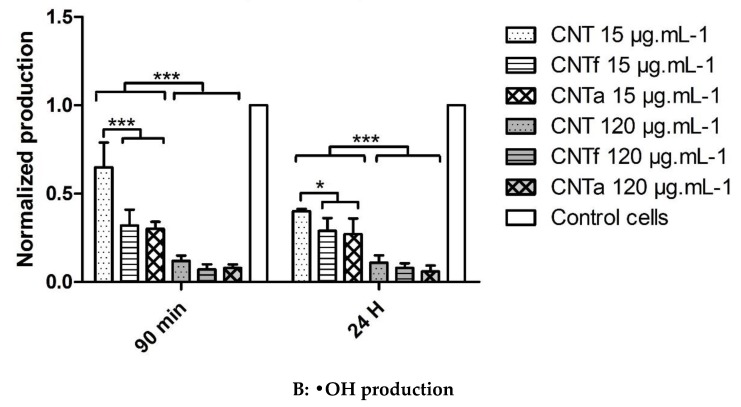
Specific O_2_^•−^ (**A**) and •OH (**B**) cytometric quantification expressed as a normalized ratio of the mean of fluorescence of treated versus untreated cells, for the three categories of MWCNTs (CNT, CNTf, and CNTa) at 15 μg·mL^−1^ and 120 μg·mL^−1^, after 90 min and 24 h exposure. Only significant values for CNT types comparison between them or by dose effect have been shown (ANOVA test, * *p* ≤ 0.05, ** *p* ≤ 0.01 and *** *p* ≤ 0.001).

The production of O_2_^•−^, quantified with HE probe, specifically increased with CNTf exposure compared to untreated cells, whatever the dose and time of contact (Figure 2A, *** p ≤ 0.001). On the other side, a strong decrease of •OH, quantified with CellRox Green, was pointed out with all types of MWCNTs compared to untreated cells, even though a less significant effect was observed for pristine CNT (Figure 2B). This specific •OH reduction was dose dependent with a decrease up to 90% both at 90 min and 24 h exposure for the higher dose at 120 µg·mL^−1^ (*** p ≤ 0.001). 

### 3.2. Anti-Oxidant Activity 

**Figure 3:**
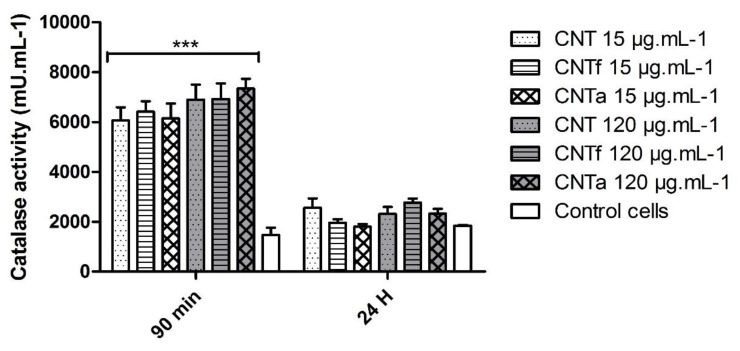
Catalase anti-oxidant activity with Amplex red kit for the three categories of MWCNTs (CNT, CNTf and CNTa) at 15 and 120 μg·mL^−1^, after 90 min or 24 h exposure (*** *p* < 0,001, ANOVA test, results standardized to those of untreated control cells).

The reduced level of total ROS observed after 24 h compared to 90 min (Figure 1) could be due to the activation of intracellular anti-oxidant systems. However, the activity of SOD did not significantly differ between MWCNT-treated cells and untreated cells (data not shown). Conversely, the catalase activity was indeed increased up to 6-fold at 90 min incubation whatever the type or dose of MWCNTs (Figure 3). However, after 24 h exposure, it came back to a level similar to that of the negative control.

### 3.3. Additional Oxidative Stress Analysis in a Cell-Free System 

#### 3.3.1. MWCNT Surface Iron Accessibility

The presence of bioavailable iron, which could be involved in free radical generation, was evaluated by incubating MWCNTs with ferrozine to assess the presence of Fe^2+^, or ferrozine and ascorbic acid as a reducing agent to quantify the total accessible iron (Fe^2+^ and Fe^3+^). Pristine CNT released iron in ferrozine solution with ascorbic acid only with average levels of 4.6 ± 0.1 nmol·g^−1^ after 8 h and 5.2 ± 0.05 nmol·g^−1^ after 24 h of incubation. CNTa and CNTf released no iron, confirming that catalytic impurities have been removed during these two-surface treatments.

#### 3.3.2. Scavenging Activity toward Hydroxyl Radicals •OH

**Figure 4:**
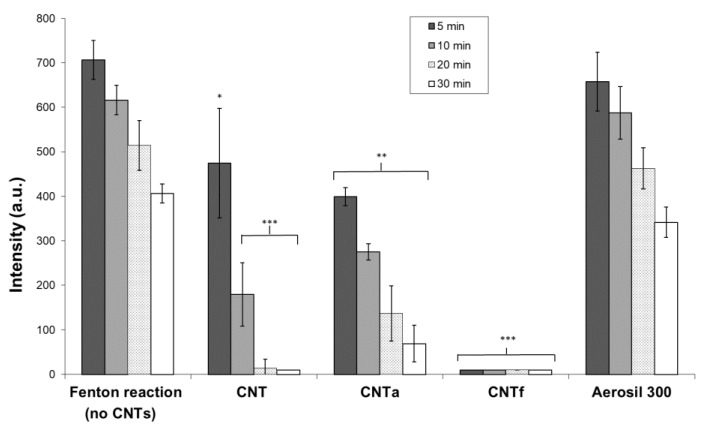
Scavenging capacity of •OH analysis by measuring the DMPO/OH adducts levels after 5, 10, 20, and 30 min with or without MWCNTs or internal control Aerosil 300. ANOVA test, comparison with the negative control (Fenton reaction), * *p* ≤ 0.05, ** *p* ≤ 0.01 and *** *p* ≤ 0.001.

In order to determine if the decrease of •OH level observed with FCM analysis (Figure 2B) could be due to a potential scavenging activity of MWCNTs, the latter was assessed in a cell-free system by the EPR spin-trapping technique (Figure 4). •OH, generated by Fenton reaction, was monitored by measuring the intensity of the spectrum of DMPO/OH• adducts. This system produced large amounts of •OH that progressively decreased over time. After 30 min of incubation, the amount of •OH was reduced by about half without MWCNTs. With MWCNTs, the signal was almost completely suppressed after 30 min. The fastest disappearance of •OH, after 5 min only, was observed for CNTf. For pristine CNT, the signal was approximately decreased by two-thirds after 10 min and disappeared after 20 min. For CNTa, the signal was lowered of by one-quarter after 10 min incubation, and it was still detectable after 30 min. Thus, the scavenging activity of pristine CNT seemed to be increased by the acid functionalization and reduced by the annealing process. An easy parallel could be done with the increase and reduction of the surface defects by these treatments. The test was repeated using Aerosil 300, and the signals were equivalent to those of the negative controls. Thus, no interferences were detected.

### 3.4. Cytotoxicity and Genotoxicity Analysis by FCM

#### 3.4.1. Death rate: PI Permeability

**Figure 5:**
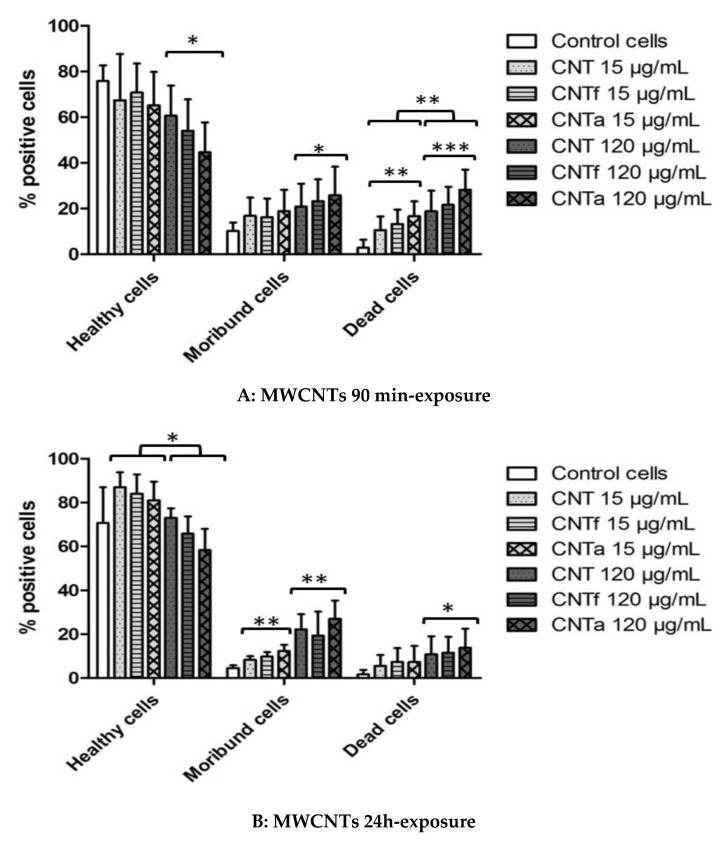
Cytotoxicity evaluation after 90 min (**A**) and 24 h (**B**) exposure to MWCNTs: percentage of dead cells (PI++), moribund cells (PI+), and healthy cells (PI−) after exposure to CNT, CNTf, and CNTa at 15 μg·mL^−1^ and 120 μg·mL^−1^. ANOVA test, comparison with the negative control (untreated cells) and by dose effect have been shown (* *p* ≤ 0.05, ** *p* ≤ 0.01 and *** *p* ≤ 0.001).

Gating of three populations of interest was achieved after PI staining: viable healthy cells (PI−), intermediate moribund cells (PI+), and dead cells (PI++) after 90 min and 24 h exposure to MWCNTs (Figure 5). After 90 min exposure to CNTa at 120 μg·mL^−1^, 30% of cells were dead (≤10% for untreated cells), and up to 25% were moribund (10% for untreated cells). After 90 min, the percentage of moribund and dead cells was 10–15% higher with 120 μg·mL^−1^ than with 15 μg·mL^−1^ of any of the three MWCNTs types. After 24 h exposure, the percentage of dead cells slightly decreased compared to the 90 min time-point, but the moribund population remained still significantly higher when compared to untreated cells. This phenomenon is probably due to the long-term mechanisms of elimination of dead cells into culture medium. Overall, a dose-dependent cytotoxicity was observed to follow MWCNTs exposure even at early time.

#### 3.4.2. Mitochondrial Membrane Potential Analysis

**Figure 6:**
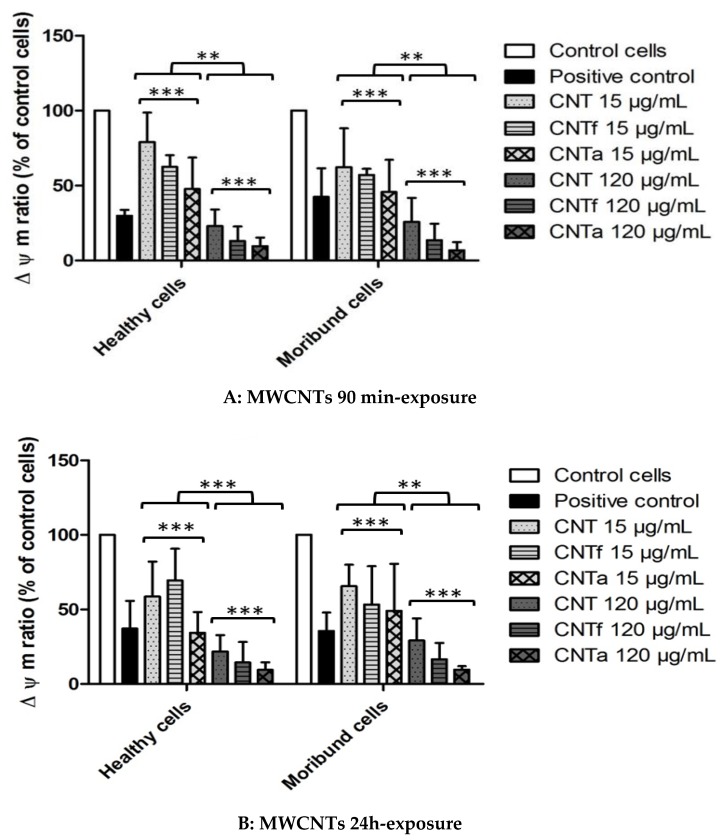
Mitochondrial membrane potential (∆Ψm) analysis after 90 min (**A**) and 24 h (**B**) exposure to MWCNTs. Ratio of DiOC6 mean fluorescence of MWCNTs treated cells on untreated control cells after 90 min or 24 h exposure to 15 μg·mL^−1^ or 120 μg·mL^−1^ of CNT, CNTf and CNTa. Healthy cells (PI−, left side) and moribund cells (PI+, right side). ANOVA test, comparison with the negative control (untreated cells) and by dose effect have been shown. * *p* ≤ 0.05, ** *p* ≤ 0.01 and *** *p* ≤ 0.001). CCCP was used as positive control. PI: propidium iodide.

A bi-parametric test for vital mitochondrial membrane potential (∆Ψm) was conducted using a DiOC6 fluorescent probe both on the healthy (PI−) and moribund (PI+) cells population, after the exclusion of all dead cells (PI++) (Figure 6). After 90 min and 24 h exposure to both healthy and moribund cells, the highest MWCNTs dose (120 μg·mL^−1^*)* induced a dramatic collapse of mitochondrial potential of up to 90% for CNTa. This mitochondrial depolarization was even higher than the one obtained for the CCCP positive control. The same tendency was observed at lower dose (15 μg·mL^−1^), but it was associated to a weaker mitochondrial collapse up to 50% with CNTa. Overall, this dose-dependent deleterious mitochondrial function was higher for CNTa than CNTf, which itself is higher than pristine CNT. 

#### 3.4.3. Genotoxicity: DNA Fragmentation and Chromatin Decondensation 

Genotoxicity was assessed by the quantification of apoptotic DNA internucleosomal fragmentation (TUNEL technique) and chromatin condensation (cell cycle analysis by PI staining). No significant DNA strand breaks were detected after 24 h of exposure with all MWCNTs and for any doses (<1% of DNA fragmentation, compared to a 47 ± 2% mean percentage DNA fragmentation observed for the positive control test, data not shown). RAW264.7 cell cycles were analyzed based on DNA staining with PI and categorized into three phases (G0/G1, S, G2+M). The coefficients of variation for the G0/G1 peaks (CV) were collected. This CV value is directly proportional to PI DNA accessibility, and in the case of a chromatin decondensation involvement, it was increased and associated to a modification of the Gaussian distribution [79].

**Figure 7:**
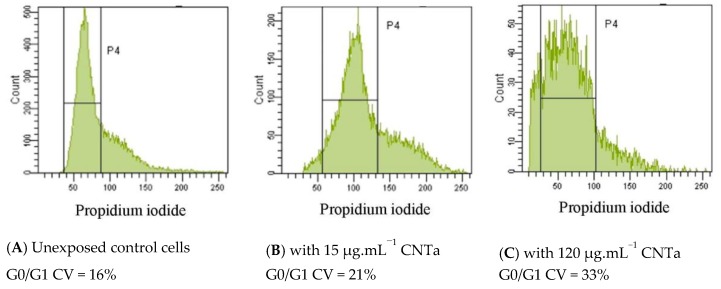
Genotoxicity evaluation with PI chromatin decondensation analysis by flow cytometry (FCM). Example of obtained results after 24 h exposure to 15 μg·mL^−1^ and 120 μg·mL^−1^ of CNTa. (**A**) Histograms of control cell cycle as G0/G1, S/G2+M phases; (**B**) Weak enlargement of G0/G1 CV peak after exposure to 15 μg·mL^−1^ CNTa (P4 area window), (**C**) Stronger enlargement of G0/G1 CV peak after exposure to 120 μg·mL^−1^ CNTa.

Figure 7 displays the results of one cell cycle analysis with CNTa compared to unexposed control cells. Table 2 exhibits CV ratio after 24 h of exposure to MWCNTs normalized to unexposed control cells levels. A MWCNTs dose-dependent DNA decondensation was observed, with a significant enlargement of the CV G0/G1 peak between the 15 μg·mL^−1^ and 120 μg·mL^−1^ doses. This trend was stronger for CNTf and CNTa than for pristine CNT. Even if no DNA fragmentation was observed, we thus pointed out a significant effect of MWCNTs on chromatin stability (CNTf and CNTa > pristine CNT), suggesting genotoxic processes, independent from an apoptotic pathway.

## 4. Discussion

Many studies have established that MWCNTs or SWCNTs lead to increased intracellular oxidative stress due to metallic impurities as iron transition metal, by promoting Harber–Weiss and Fenton–ROS production [12,25,26,27,31,35,81]. However, very few have compared the respective effects of acid functionalization and annealing treatments of pristine MWCNTs, aside from our previous studies [43,47,49].

In the present work, FCM analysis has shown that both pristine and treated MWCNTs were able to rapidly increase the intracellular ROS level of macrophages (ROS broad spectrum analysis by DCFH-DA probe). Significant after 90 min exposure, this production of ROS seemed to normalize after 24 h. Our observations matched with the now accepted fact that oxidative stress is a fast and transient process that fads down after several hours, which is mainly due to a rapid counterbalanced antioxidant activity [16,26,30]. Catalase activity was here indeed confirmed. Anti-oxidant functions could be a first explanation of the inconsistent ROS measurements shown in the literature after more than 4 h exposure to MWCNTs [26]. Pristine CNT characterization underlined that it contained some iron catalytic impurities: 0.19 wt % according to XPS (X-ray photoelectron spectroscopy) measurement (Table 1). The bioavailable iron measured by ferrozine assay reached only 15% of the total expected amount (5 nmol.mg^−1^ extracted from an initial iron content of 0.19 wt %). Part of the iron of pristine CNT was likely located within the nanotube; hence, not readily accessible. CNTf and CNTa purification was confirmed. Considering that the ROS broad spectrum levels of CNT were similar to CNTf and CNTa, the available surface iron content could be too scarce to induce crucial ROS production, contrary to what has been observed with iron rich-MWCNTs in the literature [31,80]. However, it could explain the slightly higher •OH level of pristine CNT compared to CNTf and CNTa. Other metallic impurities potentially contained in MWCNTs such as Al, Co, Mo, and Ni could also trigger oxidative stress [81,82], but previous characterization on our pristine CNT showed that it contained none of those in significant amounts [43,47].

CNTf were the only MWCNTs type to significantly stimulate a superoxide O_2_^•−^ production in RAW264.7 cells. We can hypothesize that this specific effect is related to the acid functionalization. Hsieh and al. showed indeed that due to acid functionalization, carbon nanotubes with carboxyl groups could be more reactive and capable of generating superoxide species in the presence of biological reducing agents such as NADH, by electron transfer mechanism, compared to raw nanotubes [83]. It is possible to envisage also that surface carboxylated groups can stimulate more particularly an intermediate reaction of the Fenton and Haber–Weiss cycle (fourth redox reaction in Figure S2), conducing to a more important O_2_^•−^ production [6,16].

EPR spin-trapping cell-free assays confirmed that the three MWCNTs types used in this study are able to scavenge •OH radicals (in order of significance: CNTf >> CNT ≥ CNTa). The scavenging activity seemed to be correlated to the level of structural defects, as the acid functionalization of CNTf led to their increase, conversely to the annealing treatment of CNTa. These results corroborated the hypothesis enunciated in our previous work [47] and in the literature [31,46,72,73,80]. In agreement with scavenging-cell free assays, a strong decrease of up to 90% of intracellular level of •OH for all MWCNTs samples has been observed in RAW macrophages, with the highest MWCNTs dose. A superimposable though transient antioxidant catalase activity could also induce an H_2_O_2_ reduction level, and thus indirectly also a down production of •OH species. This may partly counterbalance the increase of intracellular ROS as reported in the literature [31,46,72,73,80]. 

To conclude on oxidative stress, this study underlined that if the broad spectrum-ROS production was equivalent for the three MWCNTs types, the production of ROS subcategories, O_2_^•−^ and •OH, was impacted by the MWCNTs post-treatment. Causes may be found in MWCNTs-induced redox reactions (linked to acid functionalization) and scavenging capacity (linked with surface defects). FCM analysis and the EPR cell-free system represent two complementary methods to fully investigate the complex ROS generation and its modulation, with a specific focus on •OH radical, which is the most harmful specie. 

The anti-oxidant protective mechanisms and scavenging property were not sufficient to avoid a deleterious effect on cell viability and respiratory mitochondrial chain. A massive mitochondrial collapse was observed especially with CNTf and CNTa at the highest 120 μg. mL^−1^ dose (until 90% impairment), even though a majority of cells survived after 24 h exposure under a viable (near 60%) or moribund (near 20%) profiles. Further research would be required to follow the cell death kinetics beyond 24 h, in sub-chronical exposure condition, and monitor long-term mitochondrial function. Others studies on MWCNTs demonstrated likewise the involvement of an oxidative stress-associated mitochondrial impairment on different macrophage models but also bronchial or lung epithelial cells [25,27,28,35,36,38,39,81]. A very recent study from Snyder et al. [84] revealed that MWCNTs upregulated mitochondrial gene expression and decreased the oxygen consumption rate as well as the mitochondrial mass. Mitochondrial dysfunction was linked with a subsequent increased ROS production and could triggered cell death by different pathways [5,12,23,25].

Analysis of PI permeability confirmed the cytotoxicity of the three MWCNTs after 90 min and 24 h exposure, especially at the highest dose. All MCWNTs types induced mitochondrial alterations too. However, the trends were different between the MWCNTs types: CNTa induced a higher cytotoxicity and mitochondrial membrane potential alteration followed by CNTf (roughly CNTa > CNTf > CNT). Impacts on mitochondrial membrane potential were shown to be dependent on MWCNTs surface reactivity and degree of hydrophobicity. Zeinabad et al. demonstrated indeed that the hydrophobicity of MWCNTs resulted in cell membrane alterations and a preferential necrotic mode, while SWCNTs induced mitochondria damages and apoptotic death [65]. Ursini et al. compared the biological effects of pristine and –OH versus –COOH functionalized MWCNTs, emphasizing the importance of surface charge and dispersion states, which induced disparate interactions with the cell membrane, resulting in variable uptake modes and cytotoxicity [39]. Their results have confirmed previous observations on MWCNTs functionalization triggering cytotoxic effects, but with different cell death patterns than pristine MWCNTs, which was mostly dependent of their physicochemical characteristics especially surface charge and aggregation status [40,48,85]. The cytotoxicity observed here did not implicate an apoptotic process, considering the absence of an internucleosomal DNA fragmentation. Several tests for genotoxicity assessment are recommended by the OECD (Organisation for Economic Co-operation and Development) guidelines including two benchmark tests: the Comet assay (primary DNA damages) and the micronucleus test (chromosome lesions) [28,80,86]. These technics are complex, long, and only semi-quantitative. FCM cell cycle analysis allowed the quantification of chromatin stability by the collection of the CV values of G0/G1 peak [59,79]. This method revealed chromatin decondensation induced by the MWCNTs, which was slightly higher again for CNTa and CNTf. Other studies suggested that MWCNTs could reach the cell nucleus and directly interact with the DNA [21,23,28]. The physicochemical properties of MWCNTs should again affect this nano-biointeraction and hence the observed differences in genotoxicity profiles, as demonstrated in the recent study of Siegrist et al. [87].

It was shown that MWCNTs functionalization or annealing treatments have not induced a significant cell protective effect, but rather the contrary (results summarized in Table S1). This correlated our previous studies reporting that CNTf and CNTa increased the pro-inflammatory response [43,47], and other studies showing that functionalized carboxyl-MWCNT have been equivalent or even more deleterious in terms of inflammation, oxidative stress, and cytotoxicity, than pristine CNTs [36,39,40,48,85].

## 5. Conclusions

This work combined a cellular (FCM) and acellular (ferrozine assay and EPR cell-free system) experimental design that provided new conclusions about MWCNTs surface post-treatments, on the modulation of oxidative status *in toto* with linked cytotoxicity and genotoxicity.

1. First, no significant differences in large spectrum ROS production were observed between the MWCNTs types. Moreover, ROS enhancement was only detectable after short-term exposure, while over the long term (24 h), the antioxidant mechanisms such as catalase activity counterbalanced it.
2. Then, using our FCM multiparametric methodology for a more specific analysis of O_2_^•−^ and •OH production, it was discovered that acid functionalization and annealing actually affected the MWCNTs related to oxidative stress. Iron impurities, only detected in pristine CNT, were not the main cause of the oxidative stress.
3. CNTf, purified by acid functionalization, was characterized by a stronger increased scavenging capacity and an enhanced specific O_2_^•−^ production, compared to CNTa and CNT; meanwhile, CNTa, purified by annealing, showed the most reduced scavenger capacity.
4. Both acid functionalization and annealing treatment had deleterious impacts on the cytotoxicity i.e., the mitochondrial membrane impairment and cell death, and genotoxic effect i.e., chromatin decondensation (Table S1).
5. This study underlined that a full benefit–risk balance evaluation of the MWCNTs post-treatments on bio-toxicity should be carried out if a “safer by design” approach is attempted.
6. The methodology developed for this work could be relevant for acute or chronic nanotoxicological studies of other nanomaterials, and further thorough determination of surface properties impact on oxidative stress, cytotoxicity, and genotoxicity.

## Figures and Tables

**Table 1:** Main physicochemical features of the three types of MWCNTs (CNT, CNTf, and CNTa). TEM: Transmission Electronic Microscopy, FEG-SEM: Field Emission Gun Scanning Electronic Microscopy, XPS: X-ray photoelectron spectroscopy, SSA: Specific Surface Area, BET method: Brunauer–Emmet–Teller method, ICP-AES: Inductively Coupled Plasma—Atomic Emission spectroscopy. Zeta potentials and Isoelectric points were measured using a Zetasizer Nano ZS.

PHYSICOCHEMICAL CHARACTERISTICS	CNT	CNTf	CNTa
Diameter (nm) with FEG-SEM and TEM	17 ± 5	18 ± 5	17 ± 5
O (% atomic proportion) by XPS	8.7	7.7	3.5
SSA (m^2^ g^−1^) using BET method	212 ± 2	279 ± 1	209 ± 3
Catalytic iron metallic impurities (wt%) by XPS and ICP-AES	0.19	0.02	0.01
Structural defects indication (Id/Ig ratio) with Raman spectroscopy	1.18 ± 0.10	1.33	0.77 ± 0.13
Zeta potential in water (mV)	−23.3 ± 7	−28.7 ± 2.1	−36.4 ± 4.3
Isoelectric point in water (pH)	3.12 ± 0.32	2.6 ± 0.1	2.82 ± 0.02

**Table 2:** G0/G1 CV ratio after 24 h exposure to MWCNT, normalized to untreated control cells. Student’s test, comparison between 15 and 120 µg·mL^−1^, * *p* < 0.05.

	CV Ratio (mean + SD)
CNT 15 μg·mL^−1^	1.3 ± 0.2
CNT 120 μg·mL^−1^	1.5 ± 0.3 *
CNTf 15 μg·mL^−1^	1.4 ± 0.3
CNTf 120 μg·mL^−1^	1.8 ± 0.3 *
CNTa 15 μg·mL^−1^	1.4 ± 0.1
CNTa 120 μg·mL^−1^	1.9 ± 0.3 *

## Supplementary Materials

The following are available online at https://www.mdpi.com/2079-4991/10/2/319/s1, Figure S1: Oxidative stress versus antioxidant-catalyzing reactions including SOD and catalase activities, Figure S2: ROS catalyzing-Fenton and Haber-Weiss reactions, with intermediate redox reactions, Figure S3: Illustration of cytometric cell gating strategy on unexposed control cells (Fig S3A) versus RAW264.7 exposed to 120 µg·mL^−1^ CNTa (Fig S3B), Table S1: Evolution of the different biological parameters after RAW 264.7 macrophages exposure to MWCNTs.
