# Supplementary material for: Quantitative Flow Cytometric Evaluation of Oxidative Stress and Mitochondrial Impairment in RAW 264.7 Macrophages after Exposure to Pristine, Acid Functionalized, or Annealed Carbon Nanotubes
Source: Nanomaterials (Basel). 2020 Feb 13;10(2):319. doi: 10.3390/nano10020319 (PMC7075214; doi:10.3390/nano10020319)
Supplement: Supplementary file 1 [file nanomaterials-10-00319-s001.pdf]

nanomaterials

Supplementary

Quantitative Flow Cytometric Evaluation of Oxidative Stress and Mitochondrial Impairment in RAW 264.7 Macrophages after Exposure to Pristine, Acid Functionalized, or Annealed Carbon Nanotubes

Odile Sabido [1,2,3,*], Agathe Figarol [4], Jean-Philippe Klein [1,2], Valérie Bin [1,2], Valérie Forest [5], Jérémie Pourchez [5], Bice Fubini [6], Michèle Cottier [1,2], Maura Tomatis [6] and Delphine Boudard [1,2,*]

[1] Inserm U1059 SAINBIOSE, équipe DVH/PIB, Université Jean Monnet, Faculté de Médecine, F-42270 Saint-Etienne, France; odile.sabido@univ-st-etienne.fr (O.S.); delphine.boudard@univ-st-etienne.fr (D.B.); jeanphiklein@hotmail.fr (J.-P.K.); valerie.bin@univ-st-etienne.fr (V.B.); michele.cottier@univ-st-etienne.fr (M.C.)

[2] Université Lyon, F-42270 Saint-Etienne, France

[3] Centre Commun de Cytométrie en Flux, Saint-Etienne, F-42270 France; odile.sabido@univ-st-etienne.fr (O.S.)

[4] Ecole Nationale Supérieure des Mines, SPIN, CNRS: UMR 5307, LGF, F-42023 Saint-Etienne, France; agathe.figarol@gmail.com

[5] Mines Saint-Etienne, Univ Lyon, Univ Jean Monnet, INSERM, U1059 Sainbiose, Centre CIS, F-42023 Saint-Etienne, France; valerie.forest@mines-stetienne.fr (V.F.); jeremie.pourchez@mines-stetienne.fr (J.P.)

[6] Dipartimento di Chimica and 'G. Scansetti' Interdepartmental Center for Studies on Asbestos and other Toxic Particulates, Università di Torino, 10125, Torino, Italy; bice.fubini@unito.it (B.F.); m.tomatis@unito.it (M.T.)

* Correspondence: odile.sabido@univ-st-etienne.fr (O.S.); delphine.boudard@univ-st-etienne.fr (D.B.); Tel.: +33-477421441 (O.S.); +33-477421443 (ext.1471) (D.B.)

Received: 28 December 2019; Accepted: 7 February 2020; Published: 13 February 2020

nanomaterials

MDPI

Table S1. Evolution of the different biological parameters after RAW 264.7 macrophages exposure to MWCNTs.

	Pristine CNT				CNTf				CNTa			
Bio-toxicity parameters	90 min 15 *µg.mL⁻¹*	90 min 120 *µg.mL⁻¹*	24 h 15 *µg.mL⁻¹*	24 h 120 *µg.mL⁻¹*	90 min 15 *µg.mL⁻¹*	90 min 120 *µg.mL⁻¹*	24 h 15 *µg.mL⁻¹*	24 h 120 *µg.mL⁻¹*	90 min 15 *µg.mL⁻¹*	90 min 120 *µg.mL⁻¹*	24 h 15 *µg.mL⁻¹*	24 h 120 *µg.mL⁻¹*
Mitochondrial impairment	+	+++	++	+++	++	+++	+	+++	++	++++	+++	++++
Broad ROS production	++	+++	+	+	++	+++	0	0	++	+++	0	0
$O_2^{\bullet-}$ production	0	+	++	++	++++	++++	++++	++++	0	0	+	0
•OH decrease production	++	+++	+++	++++	+++	++++	+++	++++	+++	++++	+++	++++
Catalase activity	++++	++++	0	0	++++	++++	0	0	++++	++++	0	0
Dead cells (PI++)	+ +	+++	+	++	++	+++	+	++	++	+++	+	++
DNA fragmentation TUNEL	nd	nd	0	0	nd	nd	0	0	nd	nd	0	0
Chromatine decondensation	nd	nd	+	++	nd	nd	++	+++	nd	nd	++	+++
•OH scavenging activity in cell free system	+++				++++				++			

This summary focuses on viable cells after 90 min or 24 h exposure to 15 and 120 µg.mL⁻¹, of one of the MWCNTs types:

+ : lower than 30% change (up or down) compared to control unexposed cells

++ : from 30 to 60 % change (up or down) compared to control unexposed cells

+++ : over 60% change (up or down) compared to control unexposed cells

++++ : ≥ 90% change (up or down) compared to control unexposed cells

nd : not defined

In green: the most significant impacts compared to control unexposed cells

In red: MWCNTs type and dose with the most deleterious effect or specific action.

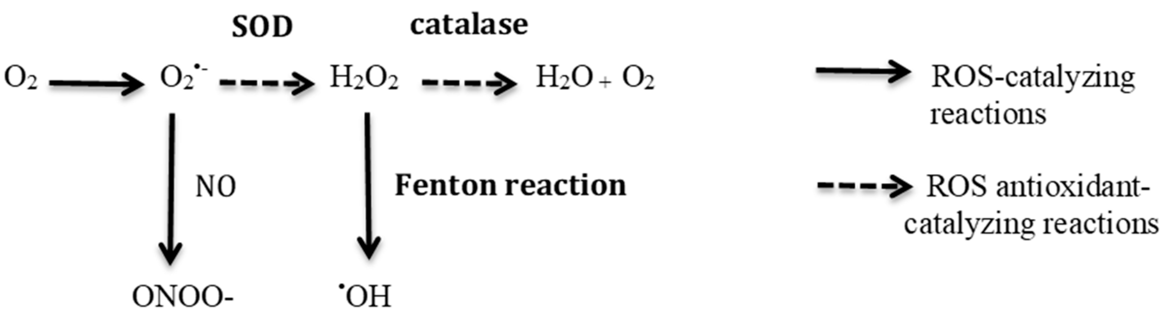

Figure S1. Oxidative stress versus antioxidant-catalyzing reactions including SOD and catalase activities.

(1) $Fe^{2+} + H_2O_2 \rightarrow Fe^{3+} + OH^- + {}^{\bullet}OH$ (Fenton reaction)

(2) $Fe^{3+} + H_2O_2 \rightarrow FeOOH^{2+} + H^+$

(3) $FeOOH^{2+} \rightarrow Fe^{2+} + {}^{\bullet}OOH$

(4) ${}^{\bullet}OOH \rightarrow H^+ + O_2^{\bullet-}$

(5) $Fe^{3+} + O_2^{\bullet-} \rightarrow Fe^{2+} + O_2$

(6) $O_2^{\bullet-} + H_2O_2 \rightarrow OH^- + {}^{\bullet}OH + O_2$ (Haber-Weiss reaction)

Figure S2. ROS catalyzing-Fenton and Haber-Weiss reactions, with intermediate redox reactions.

Fig S3A. Unexposed RAW264.7

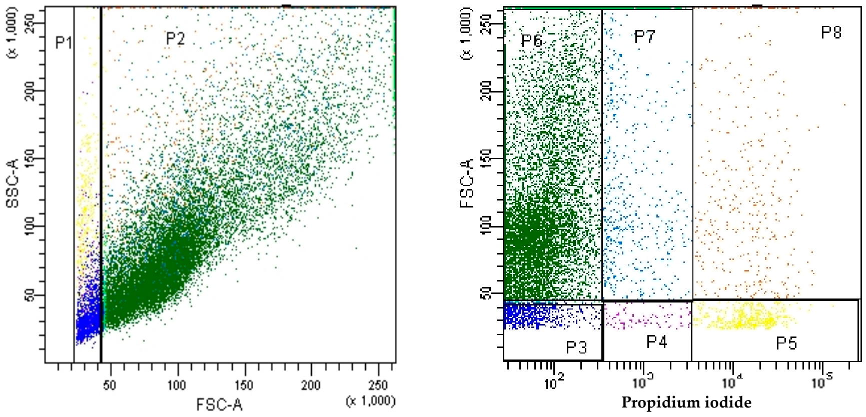

Fig S3B. RAW264.7 after CNTa 120 µg.mL^{-1} exposure

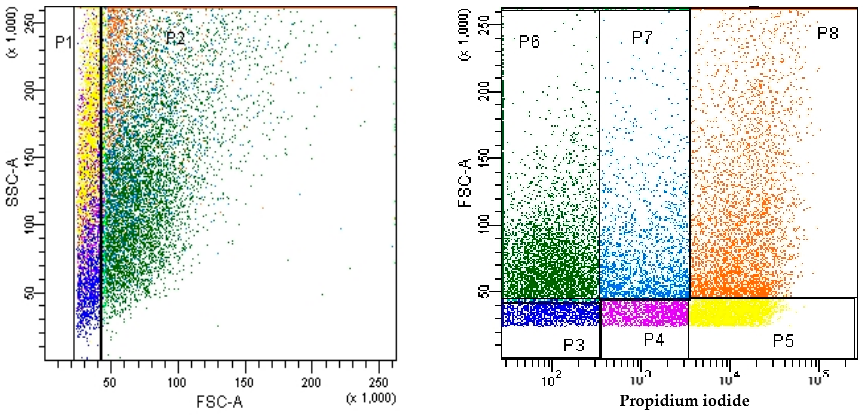

Figure S3. Illustration of cytometric cell gating strategy on unexposed control cells (Fig S3A) versus RAW264.7 exposed to 120 µg.mL^{-1} CNTa (Fig S3B). First, morphologically intact cells corresponding to usual FSC vs SSC signals elements (P2 gate) were distinguished from smaller elements with low FSC signals (P1 gate). Secondly, we draw three others gates in each of these areas, according to PI staining. In the 1st area, the P3 gate identified specifically particulates with low FSC and PI negative staining: a mix of cell debris without DNA and CNT aggregates. In the 2nd area, the P6 gate elected the viable cells (PI-); the P7 gate, an intermediate stage with moribund cells (PI+); and the P8 gate, the dead cells (PI++). According to these two types of dot plots : FSC vs SSC and FSC vs PI, this gating strategy allows to observe an evolution of the cells morphological profile as well as viability, due to contact and internalization of CNTa, associated with an increase of the P1 (CNTs agregates), P7 (moribund cells) and P8 (dead cells) sub-populations, compared to unexposed control cells.
